# Supplementary material for: Autonomous quantum heat engine based on non-Markovian dynamics of an optomechanical Hamiltonian
Source: Sci Rep. 2024 Apr 24;14:9448. doi: 10.1038/s41598-024-59881-z (PMC11043434; doi:10.1038/s41598-024-59881-z)
Supplement: Supplementary file 1 — Supplementary Information. [file 41598_2024_59881_MOESM1_ESM.pdf]

## Supplementary Material

# Autonomous Quantum Heat Engine Based on Non-Markovian Dynamics of an Optomechanical Hamiltonian

Miika Rasola and Mikko Möttönen

This Supplementary Materials contains the following figures that provide additional complementary data for the main text, but that are not pivotal for the conclusions of the work: temporal evolution of the optomechanical system free of thermal fluctuation (Fig. S(1)) dynamics of the optical and mechanical mode amplitudes in 2+1 dimensions (Fig. S(2)), dependence on the output power in the analytical model on the dissipation rates (Fig. S(3)), the dependence of the energy produced in a cycle on the mechanical-mode frequency (Fig. S(4)), and the dependence of the interaction time between the working fluid and its reservoirs on the speed of the driving mode (Fig. S(5)). Figure S(6) shows how the interaction time $\tau$ is defined for the analytical model.

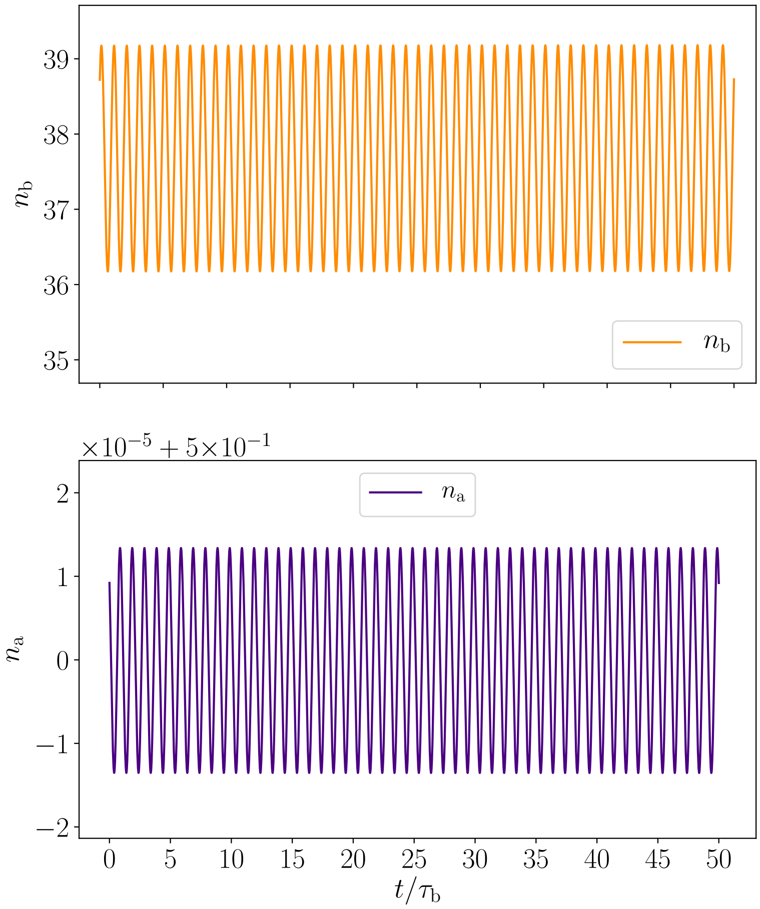


***Supplementary Figure S(1). Temporal evolution of the optomechanical system free of thermal fluctuation.*** *Temporal evolution of the mean occupation numbers of the optomechanical system modes, orange for the mechanical mode and magenta for the optical, uncoupled from the thermal noise sources. We use identical parameters values to those in Fig. 5.*

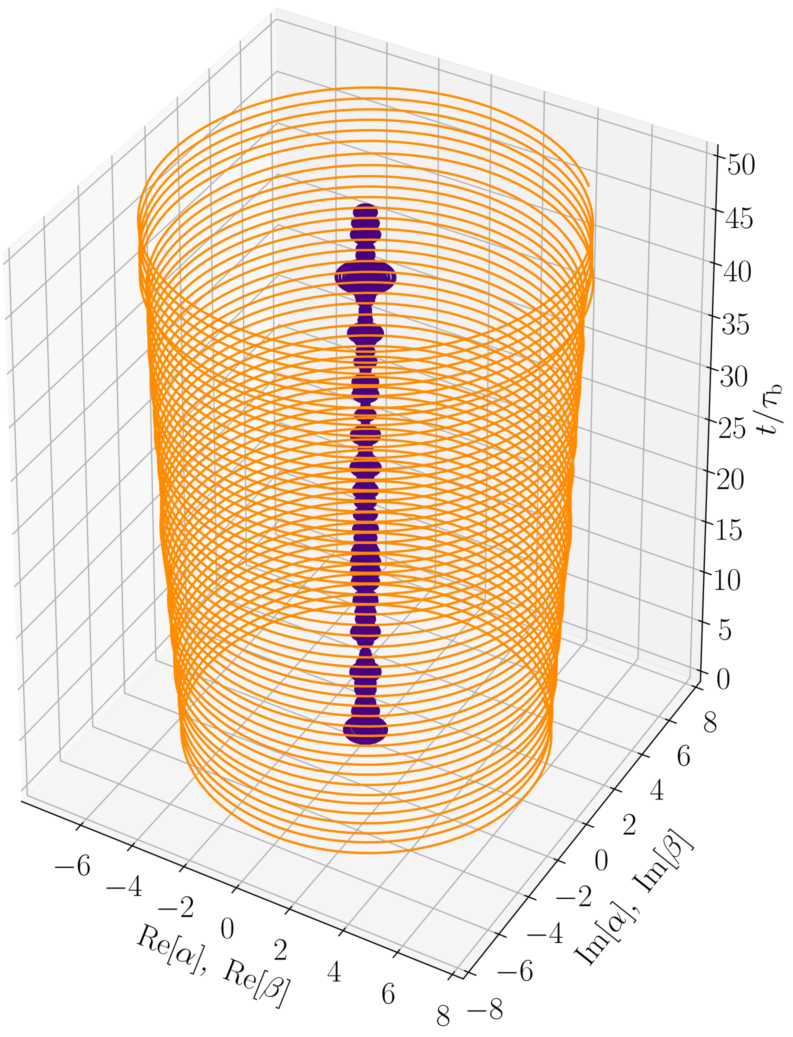


***Supplementary Figure S(2)*. Dynamics of the amplitudes of the optical and mechanical modes.** *Phase space evolution of the complex-valued amplitudes of the optical (purple color) and the mechanical (orange color) mode illustrated in two plus one dimension. These data is identical to those shown in Figs. 5(**b**) and 5(**d**).*

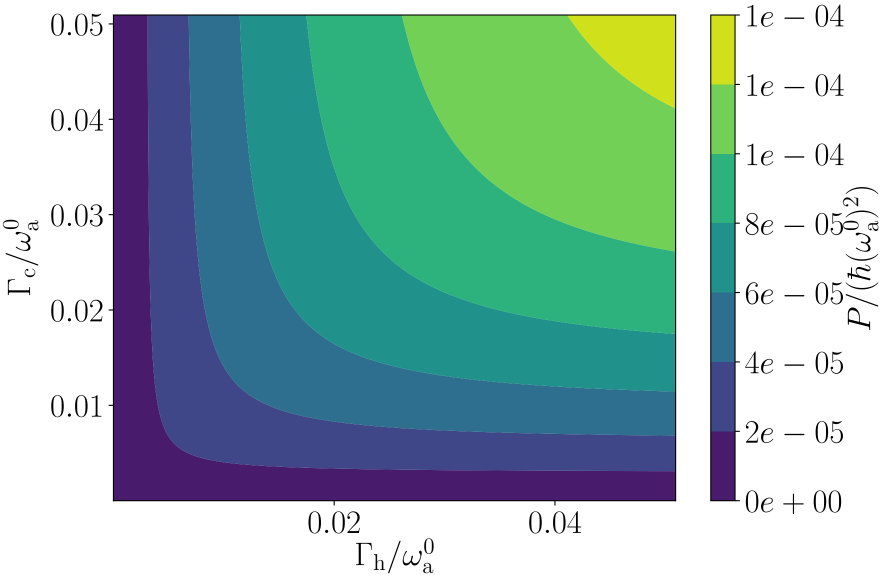


***Supplementary Figure S(3). Effect of the reservoir coupling strengths on the output power.*** *Net output power of the quantum heat engine as a function of the energy relaxation rates $\Gamma_{h/c}$ given by the analytical model. We use identical parameters values to those in Fig. 3.*

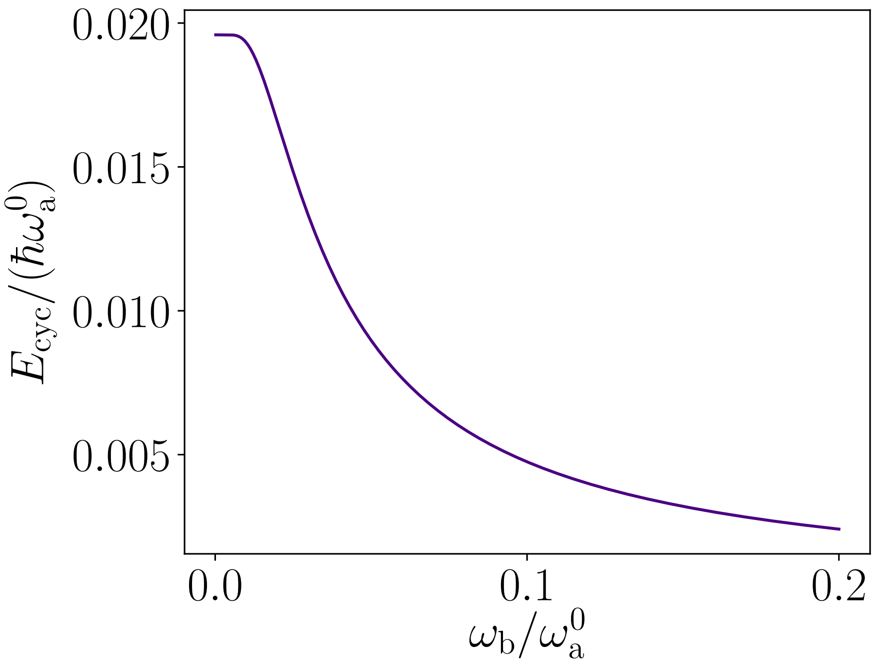


***Supplementary Figure S(4). Energy produced in a cycle.*** *Energy per cycle as given by the analytical model Eq. (5) as a function of mechanical-mode angular frequency $\omega_b$. We use identical parameters values to those in Fig. 3.*

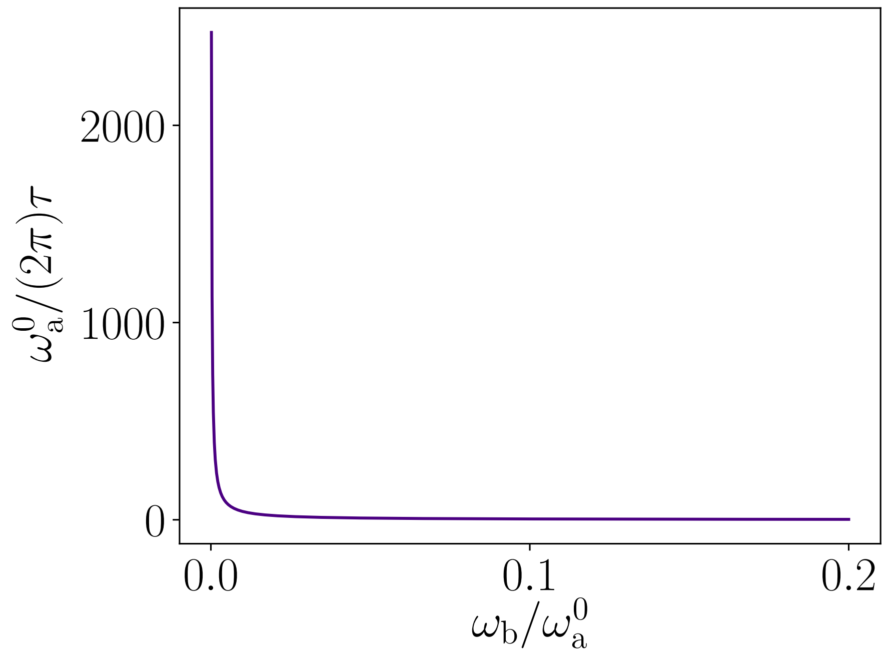


***Supplementary Figure S(5)*. Dependence of the interaction time between the working fluid and its reservoirs on the speed of the driving mode.** *Interaction time $\tau$ of the analytical model as a function of the mechanical-mode angular frequency $\omega_b$. We use identical parameters values to those in Fig. 3.*

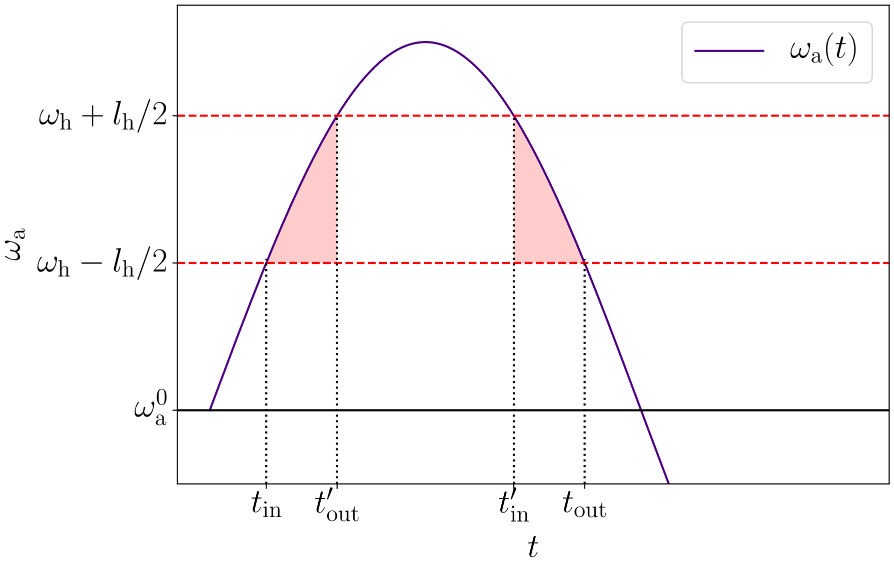


***Supplementary Figure S(6)*. Calculation of the interaction time $\tau$ of the analytical model.** *Optical-mode angular frequency $\omega_a(t)$ as a function time (purple solid line). The shaded areas represent the interaction regions of the optical mode with the hot reservoir, defining the interaction time $\tau = (t_{out} - t_{in}) - (t'_{in} - t'_{out})$, where the existence of each term in parenthesis depends on the amplitude of the angular-frequency modulation.*

The interaction time $\tau$ of the analytical model for the hot reservoir in the most general case can be calculated, as depicted in Fig., from the inequality

$$\omega_{\mathrm{h}} - l_{\mathrm{h}}/2 \leq \omega_{\mathrm{a}}^{0} + \Delta\omega_{\mathrm{a}}/2\sin(\omega_{\mathrm{b}}t) \leq \omega_{\mathrm{h}} + l_{\mathrm{h}}/2.$$

Solving the two equations arising at the limit of equality, we find the conditions

$$\begin{aligned}
t_{\mathrm{in}} &= \frac{1}{\omega_{\mathrm{b}}} \arcsin\left(\frac{2\omega_{\mathrm{h}} - l_{\mathrm{h}} - 2\omega_{a}^{0}}{\Delta\omega_{\mathrm{a}}}\right), \\
t_{\mathrm{out}} &= \frac{1}{\omega_{\mathrm{b}}} \left[\pi - \arcsin\left(\frac{2\omega_{\mathrm{h}} - l_{\mathrm{h}} - 2\omega_{a}^{0}}{\Delta\omega_{\mathrm{a}}}\right)\right], \\
t'_{\mathrm{out}} &= \frac{1}{\omega_{\mathrm{b}}} \arcsin\left(\frac{2\omega_{\mathrm{h}} + l_{\mathrm{h}} - 2\omega_{a}^{0}}{\Delta\omega_{\mathrm{a}}}\right), \\
t'_{\mathrm{in}} &= \frac{1}{\omega_{\mathrm{b}}} \left[\pi - \arcsin\left(\frac{2\omega_{\mathrm{h}} + l_{\mathrm{h}} - 2\omega_{a}^{0}}{\Delta\omega_{\mathrm{a}}}\right)\right].
\end{aligned}$$

Using these definitions, the interaction time can be calculated from $\tau = (t_{\mathrm{out}} - t_{\mathrm{in}}) - (t'_{\mathrm{in}} - t'_{\mathrm{out}})$.

The average angular frequency over the interaction period $\bar{\omega}_{\mathrm{a}}^{\mathrm{h}}$ in the analytical model is found by time averaging over the interaction period,

$$\bar{\omega}_{\mathrm{a}}^{\mathrm{h}} = \frac{1}{\tau} \int_{t_{\mathrm{in}}}^{t_{\mathrm{out}}} \left[\omega_{\mathrm{a}}^{0} + \frac{\Delta\omega_{\mathrm{a}}}{2} \sin(\omega_{\mathrm{b}}t)\right] \left[\Theta(t'_{\mathrm{out}} - t) + \Theta(t - t'_{\mathrm{in}})\right] \mathrm{d}t,$$

where $\Theta(t)$ is the Heaviside step function. Analogous calculations apply for the cold reservoir.
